# Establishing a whole blood CD4^+^ T cell immunity measurement to predict response to anti-PD-1

**DOI:** 10.1186/s12885-022-10445-2

**Published:** 2022-12-17

**Authors:** Ou Yamaguchi, Kazuyuki Atarashi, Kenichi Yoshimura, Ayako Shiono, Atsuhito Mouri, Fuyumi Nishihara, Yu Miura, Kosuke Hashimoto, Yoshiaki Miyamoto, Hitoshi Uga, Nobuo Seki, Tomoko Matsushima, Norihiro Kikukawa, Kunihiko Kobayashi, Kyoichi Kaira, Hiroshi Kagamu

**Affiliations:** 1 Division of Respiratory Medicine, Saitama Medical University International Medical Center, 1397-1 Yamane, Hidaka, Saitama 350-1298 Japan; 2 Reagent Engineering, Sysmex Corporation, 4-4-4 Takatsukadai, Nishi-ku, Kobe, Hyogo 651-2271 Japan; 3 Medical Center for Translational and Clinical Research, Hiroshima University, Hiroshima, Japan; 4 Central Research Laboratories, Sysmex Corporation, Kobe, Hyogo Japan; 5 Strategic Technology Planning, Sysmex Corporation, Kobe, Hyogo Japan

**Keywords:** Biomarkers, Effector CD4^+^ T cells, Flow cytometry, PD-1 blockade therapy, Non-small cell lung cancer

## Abstract

**Background:**

Biomarkers that can accurately predict the efficacy of immune checkpoint inhibitors (ICIs) against programmed death 1 (PD-1) ligand in cancer immunotherapy are urgently needed. We have previously reported a novel formula that predicts the response to treatment with second-line nivolumab with high sensitivity and specificity in patients with non-small cell lung cancer (NSCLC) previously treated with chemotherapy. The formula was based on the percentages of CD62L^low^CD4^+^ T cells (effector T cells; %Teff) and CD4^+^CD25^+^FOXP3^+^ T cells (regulatory T cells; %Treg) in the peripheral blood before treatment estimated using the peripheral blood mononuclear cell (PBMC) method. Here, we investigated the applicability of the formula (K-index) to predict the response to treatment with another ICI to expand its clinical applicability. Furthermore, we developed a simpler assay method based on whole blood (WB) samples to overcome the limitations of the PBMC method, such as technical difficulties, in obtaining the K-index.

**Methods:**

The K-index was evaluated using the PBMC method in 59 patients with NSCLC who received first-line pembrolizumab treatment. We also assessed the K-index using the WB method and estimated the correlation between the measurements obtained using both methods in 76 patients with lung cancer.

**Results:**

This formula consistently predicted the response to first-line pembrolizumab therapy in patients with NSCLC. The WB method correlated well with the PBMC method to obtain %Teff, %Treg, and the formula value. The WB method showed high repeatability (coefficient of variation, < 10%). The data obtained using WB samples collected in tubes containing either heparin or EDTA-2K and stored at room temperature (18–24 °C) for one day after blood sampling did not differ. Additionally, the performance of the WB method was consistent in different flow cytometry instruments.

**Conclusions:**

The K-index successfully predicted the response to first-line therapy with pembrolizumab, as reported earlier for the second-line therapy with nivolumab in patients with NSCLC. The WB method established in this study can replace the cumbersome PBMC method in obtaining the K-index. Overall, this study suggests that the K-index can predict the response to anti-PD-1 therapy in various cancers, including NSCLC.

**Supplementary Information:**

The online version contains supplementary material available at 10.1186/s12885-022-10445-2.

## Background

Immune checkpoint inhibitors (ICIs) targeting the anti-programmed death 1 (PD-1) ligand have shown epoch-making effects in several cancers, especially in terms of long-term survival [1–8]. However, a considerable number of patients do not achieve long-term antitumor efficacy because of the wide variations in their pre-existing antitumor immune status [1, 2, 4, 9–13]. Therefore, there is an urgent need to develop effective biomarkers that can accurately predict responses to anti-PD-1 therapies to ensure their appropriate clinical use [14].

In a previous study, we demonstrated that CD4^+^ T cell immunity in the peripheral blood before therapy predicted the antitumor efficacy of second-line nivolumab therapy in patients with non-small cell lung cancer (NSCLC). Furthermore, we developed a formula to predict non-responders to nivolumab based on the percentage of CD62L^low^CD4^+^ and CD4^+^CD25^+^FOXP3^+^ T cells in CD4^+^ T cells of the peripheral blood, which demonstrated a sensitivity and specificity of 85.7 and 100%, respectively [15]. The formula value predicted long-term responders with progression-free survival (PFS) of > 500 days, referred to as the K-index in this study. The study indicated that the formula value could be potentially applied as a biomarker to predict response to anti-PD-1 therapies.

Owing to phase III clinical trials, such as Keynotes 024 and 042, the initial standard therapy for patients with advanced NSCLC with PD-L1 positive tumors has shifted from cytotoxic agents to PD-1 inhibitors [10, 16]. The long-term follow-up analysis of Keynote 024 showed that more than 30% of patients with advanced NSCLC with a PD-L1 tumor proportional score (TPS) ≥ 50% who received initial treatment with pembrolizumab achieved a 5-year survival. Therefore, in this study, we first investigated whether the K-index could predict the antitumor efficacy of first-line pembrolizumab therapy in patients with advanced NSCLC with a PD-L1 TPS of ≥50%.

To date, accurate analysis of T cell subset frequencies requires complicated experimental procedures of peripheral blood samples using flow cytometry (FCM) to obtain the K-index [15] Peripheral blood mononuclear cells (PBMCs) are separated from the blood samples over a Ficoll gradient, frozen at − 80 °C, and then transferred into a liquid nitrogen tank within a week for preservation. After thawing, the frozen cells are incubated in a culture medium for 32–48 h to restore molecular expression before FCM analysis. This method is known as the PBMC method. In our previous study, we applied this PBMC method to stably analyze specimens collected at various facilities and over different periods because the results were similar to those obtained at the time of collection. However, the PBMC method is not suitable for widespread clinical testing, such as in vitro diagnostics (IVD), because of the additional time required for culture and the complexity of rapid PBMC isolation and liquid nitrogen storage. Although it is crucial to maintain the initial expression of CD62L for IVD use, CD62L is rapidly lost by enzymatic degradation at the membrane-proximal site in T cells that are not isolated from PBMCs and stored as whole blood (WB) [17] Therefore, the present study also aimed to develop a method to obtain the K-index directly from WB without cumbersome procedures. Moreover, we analyzed the analytical performance of the WB method and compared it with that of the PBMC method. Our results revealed that the WB method correlated well with the PBMC method. This study confirms the wide applicability of the K-index in clinical laboratory settings.

## Materials and methods

### Clinical samples and determination of pembrolizumab therapy clinical efficacy

**Fig. 1:**
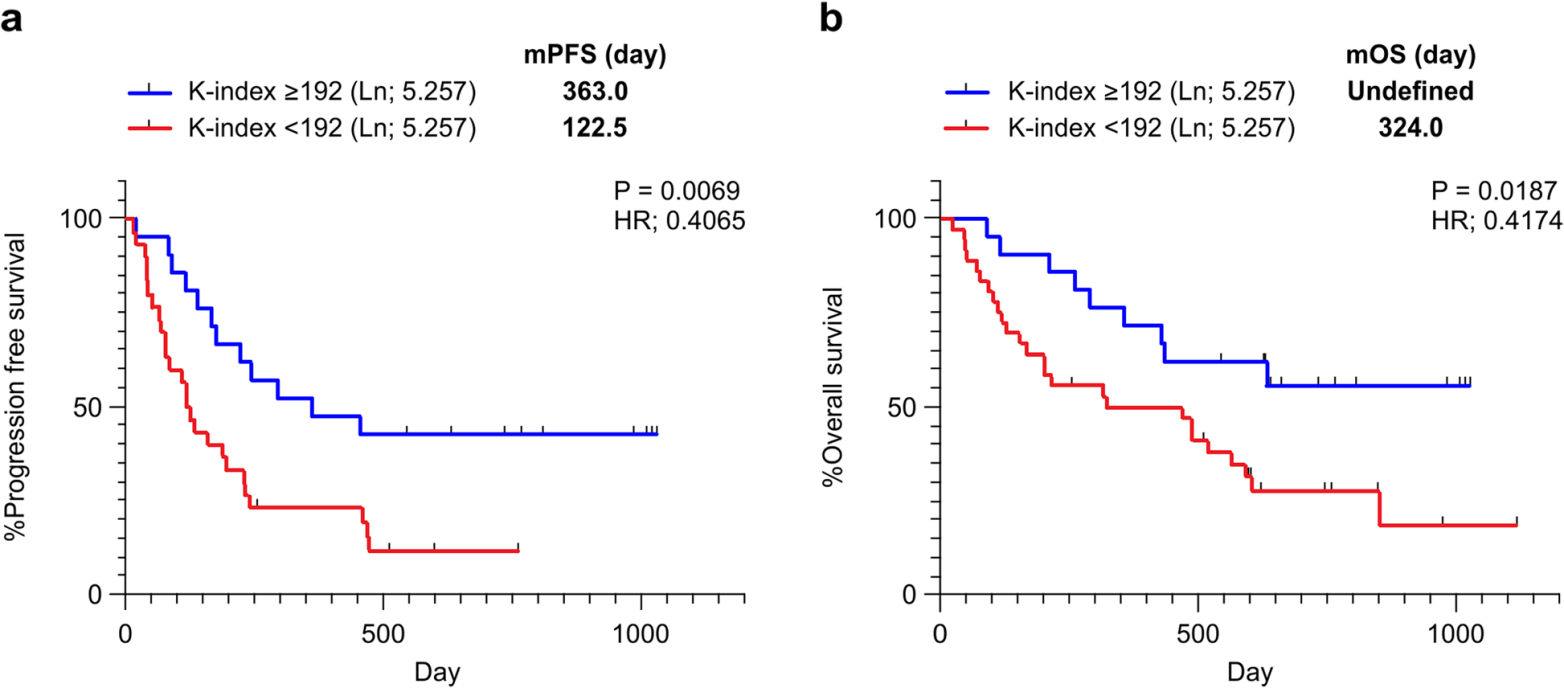
Correlation of the K-index with response to initial pembrolizumab therapy in patients with NSCLC. (**a**) PFS curves of patients receiving the first-line treatment. The blue line indicates K-index ≥192, and the red line indicates a K-index < 192. (**b**) OS curves of patients receiving first-line treatment. Statistical significance was assessed using the log-rank test. NSCLC, non-small cell lung cancer; OS, overall survival; mOS, median OS; PFS, progression-free survival; mPFS, median PFS

Fifty-nine consecutive patients with NSCLC who received fist-line pembrolizumab treatment between March 2017 and February 2019 at the Saitama Medical University International Medical Center (Saitama, Japan) were included in this study. This study was approved by ethical committee of Saitama Medical University International Medical Center. Written informed consent was obtained from all patients before sample collection, in accordance with the Declaration of Helsinki. One patient was excluded from all survival analyses because of a change in the pathological diagnosis after enrollment. Eight patients were excluded from the PFS analysis because the antitumor effect after pembrolizumab treatment could not be evaluated (Fig. 1a; Table 1). Informed consent was obtained from all participants prior to pembrolizumab treatment and blood samples were collected. K-index analysis was performed using the same procedure as previously reported [15]. Patients received a 200 mg dose of pembrolizumab every 3 weeks. Tumor response was assessed using the Response Evaluation Criteria in Solid Tumors (RECIST) version 1.1. The cutoff date for data collection was May 19, 2020. PD-L1 expression was assessed in formalin-fixed tumor samples at SRL Inc. (Tokyo, Japan) using an immunohistochemistry (IHC) assay, PD-L1 IHC 22C3 pharmDx assay (DAKO, Glostrup, Denmark). For clinical diagnosis, tumor samples were obtained via core needle biopsy, excisional biopsy, or endobronchial ultrasound-guided transbronchial needle aspiration.

**Table 1:** Patient Characteristics

Patient characteristics	*n* = 59
Age, years	
Median	69
Range	47–86
Sex, n (%)	
Male	50 (84.7)
Female	9 (15.3)
Histology, n (%)	
Adeno	26 (44.1)
Squamous	16 (27.1)
NOS	17 (28.8)
Smoking history, n (%)	
Current or former smoker	52 (88.1)
Never smoked	7 (11.9)
Disease stage, n (%)	
C-stage IIIB	5 (8.5)
C-stage IV	41 (69.5)
Recurrence	13 (22.0)
PD-L1 TPS, n (%)	
50% or greater	57 (96.6)
1–49%	2 (3.4)
Best response, n (%)	
CR or PR	21 (35.6)
SD	17 (28.8)
PD	12 (20.3)
NE	9 (15.3)

C-stage, clinical stage; CR, complete response; NE, not evaluable; NOS, not otherwise specified; PD, progressive disease; PR, partial response; SD, stable disease

### Clinical samples for establishing the WB method

Blood samples were collected from 76 patients with lung cancer at the Saitama Medical University International Medical Center (Saitama, Japan) from October 2020 to December 2021. This study was approved by ethical committee of Saitama Medical University International Medical Center. Written informed consent was obtained from all patients before sample collection, in accordance with the Declaration of Helsinki.

Blood samples were collected into heparinized CPT Vacutainer tubes (BD Biosciences, Franklin Lakes, NJ, USA) for PBMC isolation. Both heparin- and EDTA-2K-containing blood collection tubes were used to collect WB, and the samples were stored at room temperature (18–24 °C) before sample preparation for FCM.

### Sample preparation for FCM

For the PBMC method, blood samples collected in heparinized CPT Vacutainer tubes were centrifuged at 1500×*g* for 20 min at room temperature (18–24 °C) to separate PBMCs from erythrocytes and granulocytes over a Ficoll gradient. The PBMCs were then frozen at − 80 °C in Cellbanker2 (Nippon Zenyaku Kogyo Co., Ltd., Fukushima, Japan) and transferred into a liquid nitrogen tank within a week. For T cell subset analyses, cells were incubated for 32–48 h in a culture medium comprising RPMI1640 and 10% fetal calf serum (FCS) before staining.

For the WB method, CD235ab-biotin (Clone HIR2; BioLegend, San Diego, CA, USA) and streptavidin magnetic beads (BioLegend) were added to the blood samples collected in heparin or EDTA-2K-containing blood collection tubes. Red blood cells (RBCs) were removed using cell separation magnets.

Cultured PBMC- and RBC-free WB cells were stained with CD4-FITC (Clone MEM-241; EXBIO Praha, a.s., Vestec, Czech Republic), CD25-PECy7 (Clone MEM-181, EXBIO), and CD62L-APC (Clone LT-TD180, EXBIO) at room temperature (18–24 °C). FOXP3 was then stained with a Foxp3/Transcription Factor Staining Buffer set (Thermo Fisher Scientific, Waltham, MA, USA) and anti-FOXP3-PE (Clone PCH101, Thermo Fisher Scientific or Clone 206D, BioLegend) according to the supplier’s protocol. The optimal dilution ratio of the antibodies and incubation time were determined in a pilot study and ensured in this study. The antibody panel in this study was confirmed to have the same performance as that of the panel in our previous study [15] (Supplementary Fig. 1).

### Flow cytometry

The samples were measured using BD Accuri™ C6 Plus (BD Biosciences), BD FACSCanto™ II (BD), or Sysmex XF-1600 (Sysmex Corporation, Kobe, Japan) according to the manufacturer’s instructions. Fifteen thousand (15,000) CD4^+^ cells were analyzed in each measurement. The data were analyzed using FlowJo software version 10 (FlowJo, Ashland, OR, USA).

### K-index calculation

The K-index was calculated using the prediction formula reported in a previous study [15]. The formula X^2^/Y was based on the percentages of CD62L^low^ cells (X) and CD25^+^FOXP3^+^ cells (Y) in the total population of CD4^+^ cells.

To evaluate the correlation of the K-index with the PBMC method, the formula was slightly modified from that reported in our previous study. In this study, we calculated the natural logarithm of X2/Y.

### Statistical analysis

The data in Fig. 1 and Supplementary Fig. 2 were statistically analyzed at Saitama Medical University using Prism 9 (GraphPad Software, San Diego, CA, USA). Survival curves were estimated using the Kaplan-Meier method. All *P*-values were two-sided, and statistical significance was set as *P* < 0.05.

The data in Figs. 4–7 were statistically analyzed at Sysmex Corporation using StatFlex (Artech, Osaka, Japan). A correlation analysis was performed using a linear regression model.

## Results

### K-index to predict pembrolizumab response

As previously mentioned, the index obtained by dividing the square of the CD62L^low^ CD4^+^ T cell ratio by the CD4^+^CD25^+^FOXP3^+^ T cell ratio, analyzed using pretreatment peripheral blood, can be used to predict the response to nivolumab therapy in patients with previously treated NSCLC [15]. In this study, we prospectively evaluated whether the index obtained from pretreatment peripheral blood can be used as an accurate prognostic indicator in 59 consecutive patients with advanced or recurrent NSCLC who received initial pembrolizumab treatment. As this was an observational study of clinical treatment, it was performed following the Japanese insurance approval criteria. All patients were PD-L1 TPS positive, and 57 of the 59 patients had TPS ≥ 50%. The characteristics of the patients included in this study are listed in Table 1.

The overall analysis showed a median PFS (mPFS) of 206 days and a 12-month PFS of 37.1%, which was similar to the subgroup analysis of KEYNOTE-042 [16], which reported an mPFS of 207 days and a 12-month PFS of 37.1% in patients with a TPS ≥ 50% (Supplementary Fig. 2a and b). Moreover, PFS and overall survival (OS) were analyzed in the two groups using the index obtained from the analysis of pretreatment peripheral blood, and the index threshold was 192 (obtained in our previous study [15]). PFS analysis showed that the effect of pembrolizumab was significantly better in the group with an index ≥192 (hazard ratio [HR], 0.4065; *P* = 0.007 [log-rank test]). The mPFS was 363.0 days in the group with an index ≥192 and 122.5 days in the group with an index < 192 (Fig. 1a). OS was also significantly better in the group with an index ≥192; the median OS (mOS) was undefined in the group with index ≥192 and 324.0 days in the group with index < 192 (HR, 0.417; *P* = 0.19; Fig. 1b). These results indicate that our formula consistently predicted the response to first-line pembrolizumab in patients with NSCLC.

### Development of the WB method to obtain the K-index

**Fig. 2:**
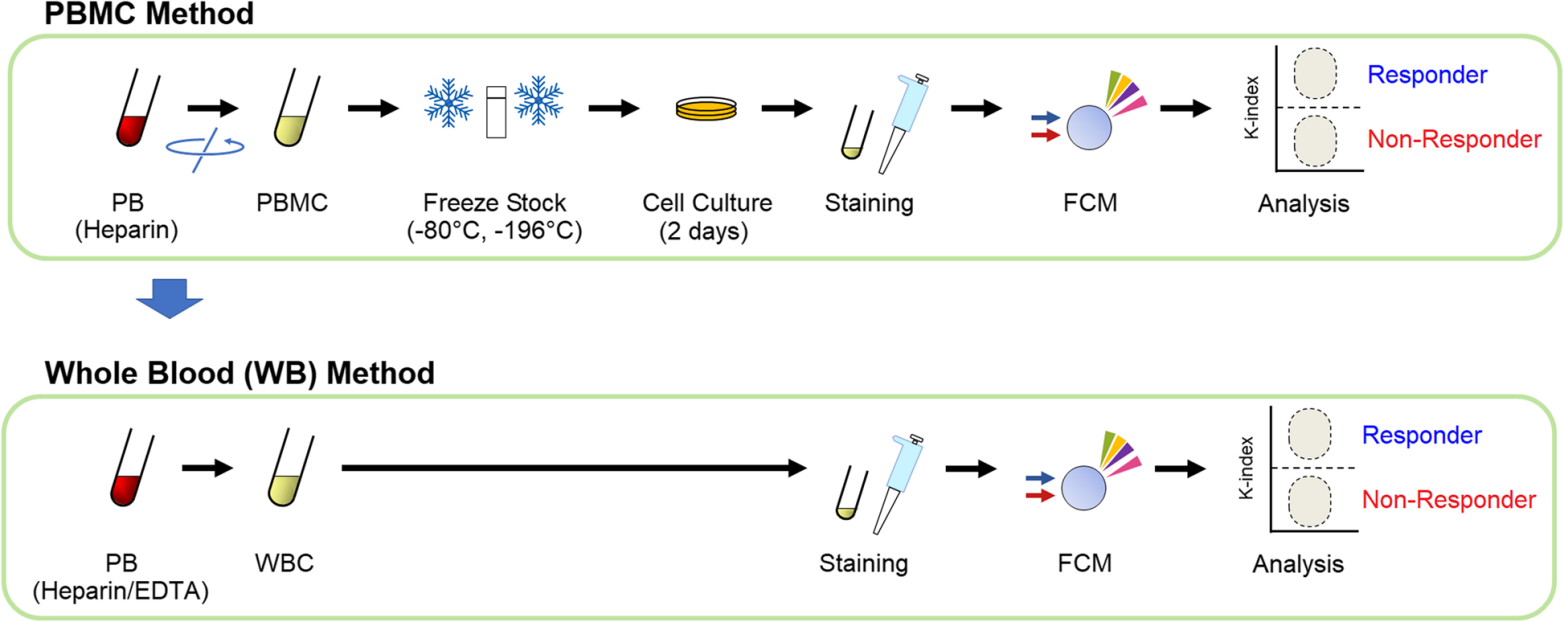
Schematic representation of the PBMC and WB methods. WB, whole blood; FCM, flow cytometry; PB, peripheral blood; PBMC, peripheral blood mononuclear cell; RBC, red blood cell; WBC, white blood cell

As the K-index was useful for predicting the response to pembrolizumab, and its significance was confirmed, we aimed to develop an easier method to measure the index. An outline of the PBMC and WB methods is shown in Fig. 2. The WB method was designed for automated sample preparation.

**Fig. 3:**
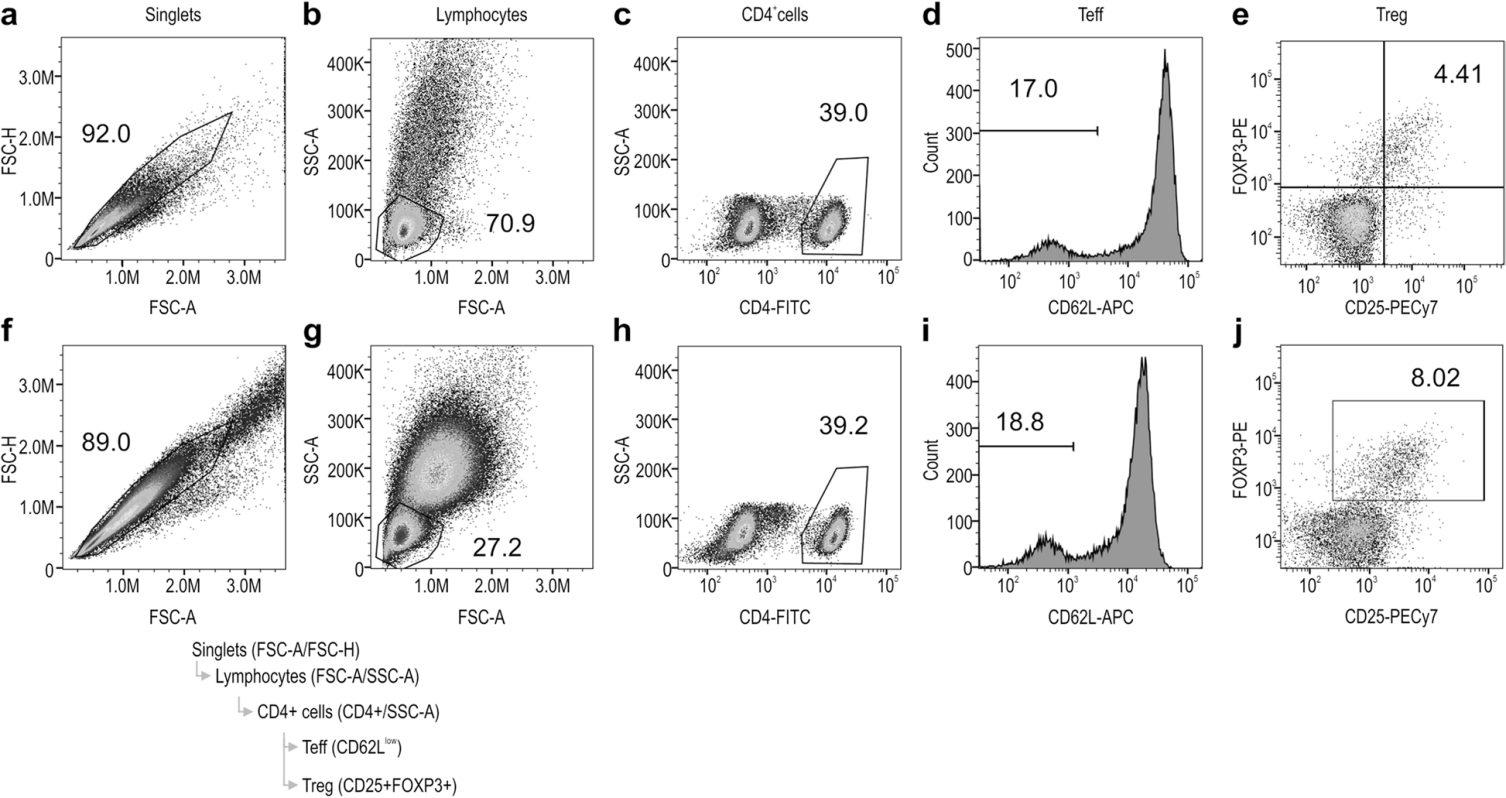
Gating strategy for FCM data analysis. **a, f** Singlet cells gated using FSC-A/FSC-H dot plot. **b, g** Lymphocytes gated using FSC-A/SSC-A dot plot. **c, h** CD4^+^T cells gated using CD4^+^/SSC-A dot plot. **d, i** Cells with low CD62L expression are selected as CD4^+^Teff cells. **e, j** Cells expressing both CD25 and FOXP3 are selected as CD4^+^Treg cells. FCM, flow cytometry; Teff, effector T cells; Treg, regulatory T cells

The data obtained from the FCM measurements were analyzed using the gating strategy illustrated in Fig. 3. Singlet cells were gated using an FSC-A/FSC-H dot plot (Fig. 3a and f). Lymphocytes were gated using an FSC-A/SSC-A dot plot (Fig. 3b and g). CD4^+^ T cells were gated using a CD4^+^/SSC-A dot plot (Fig. 3c and h). Cells with low CD62L expression were selected as CD4^+^ effector T cells (Teff; Fig. 3d and i). Cells expressing both CD25 and FOXP3 were selected as CD4^+^ regulatory T cells (Fig. 3e and j). Considering the future automation of WB method analysis, a rectangular fixed gate was adopted for the Treg gate setting, as shown in Fig. 3j.

**Fig. 4:**
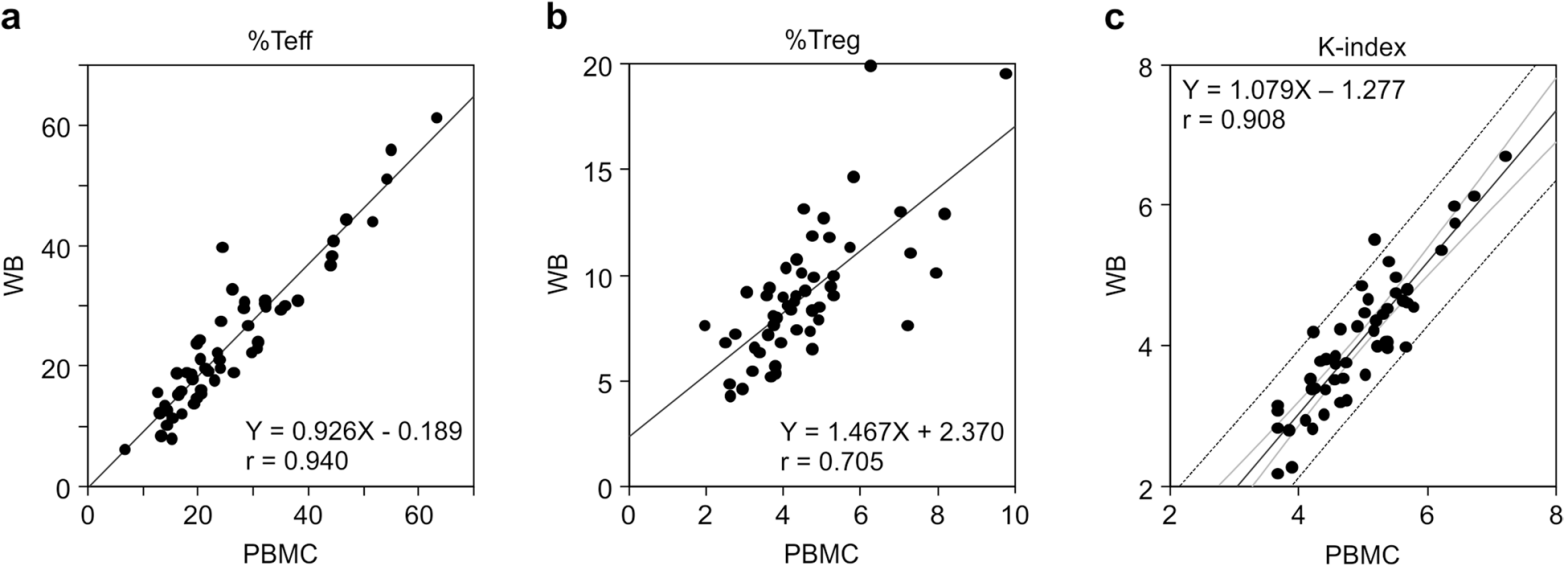
The correlation between measurements using the PBMC and WB methods. **a–c** Blood samples were collected from 53 patients with lung cancer; each sample was divided into two and measured using PBMC and WB methods. Regression lines and correlation coefficients were calculated for %Teff (**a**), %Treg (**b**), and the K-index (**c**). The gray line represents the regression line, the light gray line represents the 95% confidence interval, and the dotted line represents the 95% prediction interval. PBMC, peripheral blood mononuclear cells; Teff, effector T cells; Tregs, regulatory T cells

Moreover, we investigated the correlation between the PBMC and WB measurements. The correlation coefficients for %Teff, %Treg, and the K-index between the two methods were 0.940, 0.705, and 0.908, respectively (Fig. 4a–c). The WB method correlated well with the PBMC method, with a sufficiently high K-index correlation. However, the %Treg value in the WB method was higher than that in the PBMC method, and subsequently, the K-index value in the WB method was lower than that in the PBMC method (Fig. 4b and c). These results suggest that the cutoff value to predict the response to anti-PD-1 therapy should be adjusted in future clinical studies.

We then investigated the repeatability of the %Teff, %Treg, and K-index measurements using the WB methods. The same measurements were repeated by equally dividing each blood sample into five tubes. As shown in Table 2, the percentage coefficient of variation values of the five measurements of %Teff, %Treg, and K-index was less than 10%, indicating that the WB method had a high repeatability.

**Table 2:** Repeatability

	%Teff	%Treg	K-index
Mean ± SD	2.46 ± 1.62	3.50 ± 1.69	6.17 ± 3.78

Blood samples were collected from 40 lung cancer patients. Each sample was divided into 5 aliquots and the aliquots were processed and measured simultaneously by the WB method. The percentage coefficient of variation from each patient sample is described in this table

We confirmed that the values (%Teff, %Treg, K-index) obtained by excluding dead cells from PBMCs using CyTOF [18] were correlated with those obtained by the WB method (Supplementary Fig. 3). This result indicated no or slight influence of dead cell contamination in the measurement of the WB method established in this study.

### Adaptability of the WB method as a diagnostic test

**Fig. 5:**
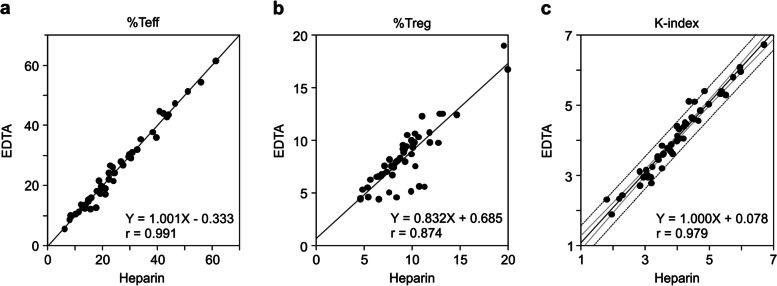
Effect of different anticoagulants during blood sampling in the WB method. **a–c** Blood samples were collected from 50 patients with lung cancer. Each sample was collected in heparin-containing and EDTA-2K-contained blood collection tubes. The correlation of the data obtained by both blood sampling methods is shown as %Teff (**a**), %Treg (**b**), and K-index (**c**). Regression lines and correlation coefficients were calculated for %Teff, %Treg, and the K-index. The gray line represents the regression line, the light gray line represents the 95% confidence interval, and the dotted line represents the 95% prediction interval. WB, whole blood; Teff, effector T cells; Treg, regulatory T cells

The anticoagulants Heparin and EDTA-2K are commonly used during blood collection for blood cell analysis [19]. We investigated whether there were differences in the measured values of %Teff, %Treg, and K-index using the WB method depending on whether heparin or EDTA-2K was used. We found that regardless of whether heparin or EDTA-2K was used at the time of blood collection, the measured values of %Teff, %Treg, and K-index were the same for the same healthy volunteers or patients with lung cancer (Fig. 5a–c). These results indicate the possibility of sharing blood samples between K-index testing and other blood tests. This will contribute to a reduction in patient burden at the time of testing.

**Fig. 6:**
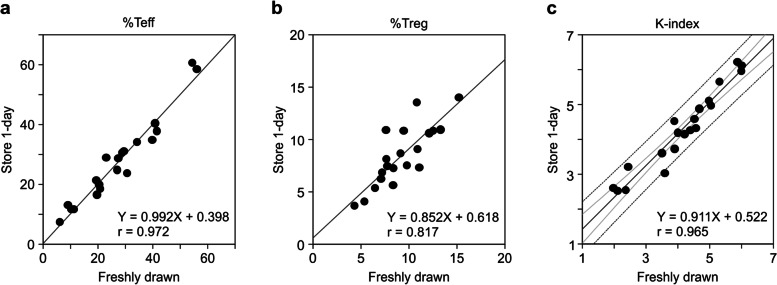
Storage stability of blood samples in the WB method. **a**–**c** Blood samples collected into heparin-containing blood collection tubes from 20 patients with lung cancer patients were measured by the WB method a day after blood collection and compared with the values measured in the same manner within 4 h after blood collection. Regression lines and correlation coefficients were calculated for %Teff (**a**), %Treg (**b**), and K-index (**c**). Twenty-two samples collected from healthy individuals were measured using the WB method a day after blood collection and compared with the values measured in the same manner within 4 h after blood collection. The gray line represents the regression line, the light gray line represents the 95% confidence interval, and the dotted line represents the 95% prediction interval. WB, whole blood; Teff, effector T cells; Treg, regulatory T cells

Depending on the circumstances, the collected blood samples were measured either immediately or at another laboratory. Therefore, we investigated whether blood samples could be stored for measurement using the WB method. Blood samples from patients with lung cancer were stored in heparin-containing blood collection tubes at room temperature (18–24 °C) for 1 d after blood sampling. The values of %Teff, %Treg, and the K-index of the same sample measured within 4 h after blood collection and after storage for 1 d (Fig. 6a–c) were the same. These results indicate that the blood samples can be stored at room temperature and sent to clinical laboratories to measure K-index.

**Fig. 7:**
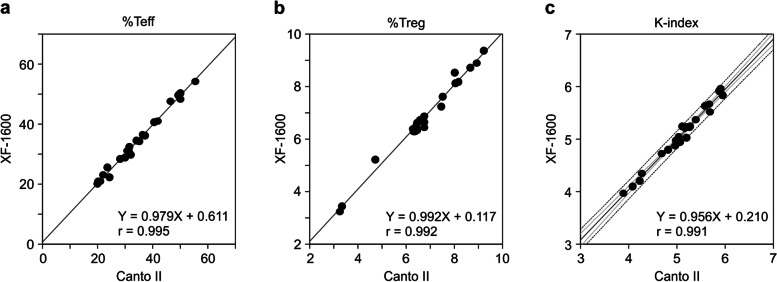
Effect of different FCM measuring devices on measured values in the WB method. **a–c** Blood samples were collected from 24 pretreated patients with lung cancer using the WB method and measured simultaneously using both XF-1600 and FACS Canto II. The correlation of both measurements was analyzed, and regression lines and correlation coefficients were calculated for %Teff (**a**), %Treg (**b**), and the K-index (**c**). The gray line represents the regression line, the light gray line represents the 95% confidence interval, and the dotted line represents the 95% prediction interval. WB, whole blood; FCM, flow cytometry; Teff, effector T cells; Treg, regulatory T cells

Finally, we examined whether the WB method can be applied to different FCM instruments. The same blood samples from patients with lung cancer prepared using the WB method were measured simultaneously using a BD FACSCanto II and Sysmex XF-1600. The measured results were well correlated in both measurements using FACSCanto II and XF-1600 (Fig. 7a–c), indicating that the measurement of the K-index is a robust test and is not affected by differences in FCM instruments.

## Discussion

Various PD-1 inhibitor combination therapies are being developed to enhance T cell restoration and increase the probability of long-term survival of patients with cancer [20–23]. However, excessive T cell restoration can lead to an increased incidence and severity of immune-related adverse events [21, 23–25]. To resolve this dichotomy, predicting whether patients will achieve sufficient long-term survival using a single PD-1 inhibitor is necessary. Monotherapy should be used when the probability of long-term survival with PD-1 blockade therapy is high, and combination therapy should be used when the probability is low [26]. Tumor PD-L1 expression assessed by IHC correlates with PD-1 inhibitor treatment efficacy and has been used as a reference for treatment selection in clinical practice [27]. However, the expression levels of PD-L1 detected by IHC lacks predictive accuracy because of its temporal and spatial heterogeneity [21, 23]. In this study, we demonstrated that among patients with NSCLC with a PD-L1 TPS ≥ 50%, despite varied pretreatment K-index, the PFS after pembrolizumab treatment was accurately predicted by the K-index. Therefore, the K-index is a promising diagnostic biomarker for predicting response to anti-PD-1 therapy.

Recent studies have revealed the main point of action of PD-1 inhibitors in CD8^+^ T cells [28, 29]. CD4^+^ T cells are essential for antitumor immunity, whereby they act as the driving force of the cancer immunity cycle by assisting the priming and clonal expansion of CD8^+^ T cells in the lymph nodes, promoting their migration into the peripheral blood circulation and infiltration into the tumor microenvironment []. Therefore, the K-index acts as a predictive biomarker that reflects the strength of CD4^+^ T cell immunity. Furthermore, it can be measured with a minimally invasive diagnostic method using peripheral blood and can also be measured over time. The K-index is a potential biomarker for appropriate patient selection in the development of new therapies using ICIs, such as adjuvant or combination therapy with ICI.

In our previous study [15], the experimental method for obtaining the K-index included many techniques requiring experimental skills that may not be suitable for widespread diagnostic testing. Therefore, in this study, we developed a method that can immediately and simply measure the K-index using WB samples. This method is based on isolating red blood cells from WB and simultaneously staining them with four types of fluorochrome-conjugated antibodies (anti-CD4, anti-CD25, anti-CD62L, and anti-Foxp3). The K-index measurements of the WB method correlated well with those of the original PBMC method (r = 0.908; Fig. 4c), and its repeatability was high (coefficient of variation < 10%; Table 2). The analytical performance and repeatability met the requirements of recent high-sensitivity FCM assay systems [31]. Therefore, the WB method can be used to predict the response to anti-PD-1 therapy instead of the PBMC method. However, since the K-index of the WB method was lower than that of the PBMC method (Fig. 4c), the cutoff value for clinical diagnosis needs to be adjusted accordingly. In this study, we conducted various experiments to develop a method that can be widely applied in clinical settings. We demonstrated that both heparin and EDTA-2K could be used as anticoagulants for blood sampling, and WB samples can be stored at room temperature (18–24 °C) for at least one day before measurement. Moreover, no difference was observed in the K-index measurements between the BD FACSCanto II and Sysmex XF-1600. Overall, here, we demonstrated the analytical performance of the WB method; however, we did not investigate its clinical performance. Therefore, clinical trials are needed to confirm the ability of the K-index (measured with the WB method) to predict the response to anti-PD-1 therapy.

We are currently working to evaluate the stability of the stored blood sample for more than 2 days and develop an automated sample preparation system for the WB method. Our future research aims to conduct multicenter studies to validate the clinical applicability of the K-index measured using the WB method and determine the cutoff values for clinical diagnosis.

## Conclusion

This study demonstrated that the K-index could predict the efficacy of various anti-PD-1 therapies. The newly established WB method for obtaining the K-index can replace the cumbersome PBMC method used in the past. The findings of this study are expected to help demonstrate the importance of the K-index in predicting the response to anti-PD-1 therapy in various cancers, including NSCLC.

## Supplementary Information

**Additional file 1: Supplementary Fig. 1.** Performance comparison between the antibody panels in the previous report [15] (X-axis) and this report (Y-axis). **(a–c)** Twenty commercially available PBMC (Cellular Technology Ltd., Shaker Heights, OH, USA) were stained with CD4-BV650 (Clone OKT4, BioLegend, San Diego, CA, USA), CD25-PE-CF594 (Clone M-A251, BD, Franklin Lakes, NJ, USA), CD62L-BV421 (Clone DREG56, BioLegend) and anti-FOXP3-PE (Clone 236A/E7, Thermo Fisher Scientific, Waltham, MA, USA) as the antibody panel in the previous report. The samples were analyzed using a BD FACSLyric™ (BD Biosciences). The correlation of the data obtained by both panels is shown as %Teff **(a)**, %Treg **(b)**, and the K-index **(c)**. Regression lines and correlation coefficients were calculated for %Teff, %Treg, and the K-index. The gray line represents the regression line, the light gray line represents the 95% confidence interval, and the dotted line represents the 95% prediction interval. PBMC, peripheral blood mononuclear cells; Teff, effector T cells; Tregs, regulatory T cells.

**Additional file 2: Supplementary Fig. 2.** Overall analysis data of response to initial pembrolizumab therapy in patients with NSCLC. Analysis of mPFS **(a)** and mOS **(b)**. mOS, median overall survival; mPFS, median progression-free survival; NSCLC, non-small cell lung cancer; PD, progressive disease; PR, partial response; SD, stable disease.

**Additional file 3: Supplementary Fig. 3.** Correlation between measurements obtained using the PBMC and WB methods. The measurements were performed using CyTOF in the PBMC method and FCM in the WB method. The gating strategy of CyTOF analysis was performed as described in a previous study [18]. In brief, dead cells were removed using 198Pt staining. The analysis of the lower layer of CD4^+^ cells was the same as the FCM analysis. Blood samples were collected from 53 patients with lung cancer. **(a–c)** Each sample was divided into two and measured using PBMC and WB methods. Regression lines and correlation coefficients were calculated for %Teff **(a)**, %Treg **(b)**, and the K-index **(c)**. The gray line represents the regression line, the light gray line represents the 95% confidence interval, and the dotted line represents the 95% prediction interval. PBMC, peripheral blood mononuclear cells; Teff, effector T cells; Tregs, regulatory T cells.

## Data Availability

All data generated or analyzed during this study are included in this published article (and its supplementary information files).

## Abbreviations

ICI: immune checkpoint inhibitor
IHC: immunohistochemistry
IVD: in vitro diagnostics
mOS: median overall survival
mPFS: median progression-free survival
NSCLC: non-small cell lung cancer
OS: overall survival
PBMC: peripheral blood mononuclear cell
PFS: progression-free survival
RBC: red blood cell
RECIST: Response Evaluation Criteria in Solid Tumors
Teff: effector T cells
FCM: flow cytometry
Treg: regulatory T cells
TPS: tumor proportion score
