# Supplementary figures and images for: Establishing a whole blood CD4+ T cell immunity measurement to predict response to anti-PD-1
Source: BMC Cancer. 2022 Dec 17;22:1325. doi: 10.1186/s12885-022-10445-2 (PMC9759885; doi:10.1186/s12885-022-10445-2)

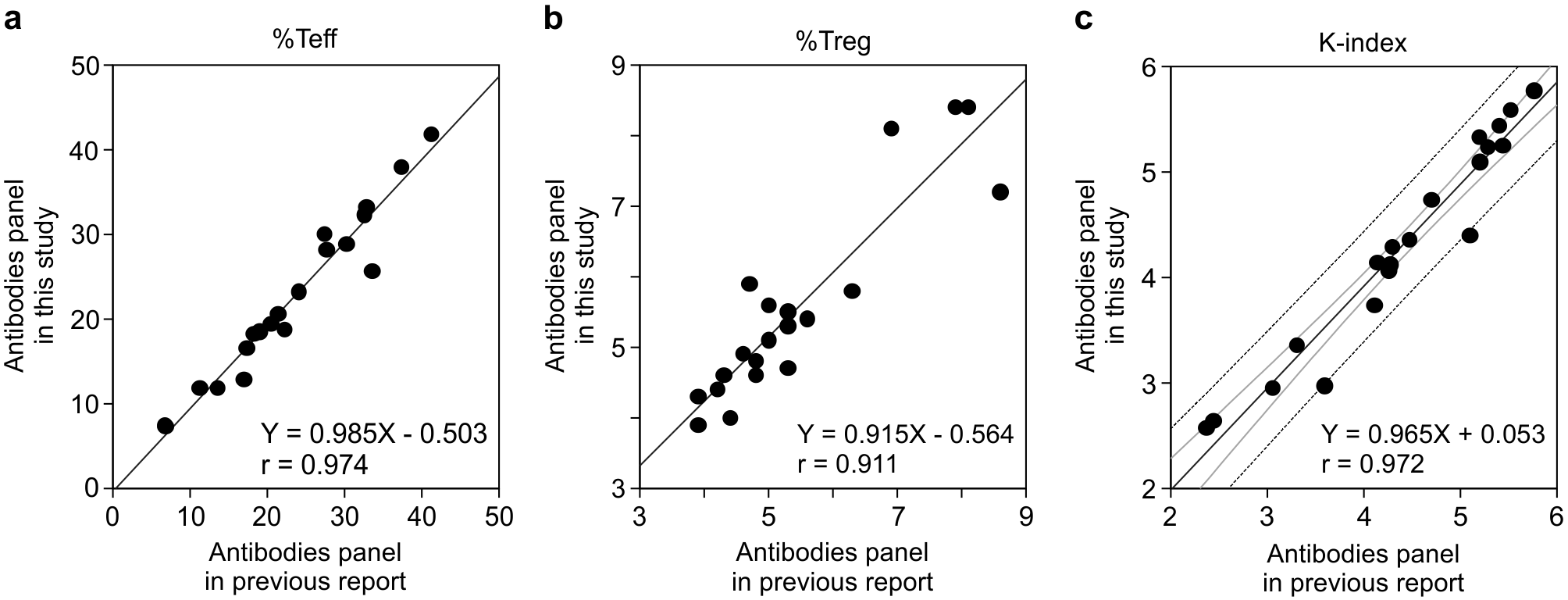

Supplement: Supplementary file 1 — Additional file 1: Supplementary Fig. 1. Performance comparison between the antibody panels in the previous report [15] (X-axis) and this report (Y-axis). (a–c) Twenty commercially available PBMC (Cellular Technology Ltd., Shaker Heights, OH, USA) were stained with CD4-BV650 (Clone OKT4, BioLegend, San Diego, CA, USA), CD25-PE-CF594 (Clone M-A251, BD, Franklin Lakes, NJ, USA), CD62L-BV421 (Clone DREG56, BioLegend) and anti-FOXP3-PE (Clone 236A/E7, Thermo Fisher Scientific, Waltham, MA, USA) as the antibody panel in the previous report. The samples were analyzed using a BD FACSLyric™ (BD Biosciences). The correlation of the data obtained by both panels is shown as %Teff (a), %Treg (b), and the K-index (c). Regression lines and correlation coefficients were calculated for %Teff, %Treg, and the K-index. The gray line represents the regression line, the light gray line represents the 95% confidence interval, and the dotted line represents the 95% prediction interval. PBMC, peripheral blood mononuclear cells; Teff, effector T cells; Tregs, regulatory T cells. [file 12885_2022_10445_MOESM1_ESM.tif]

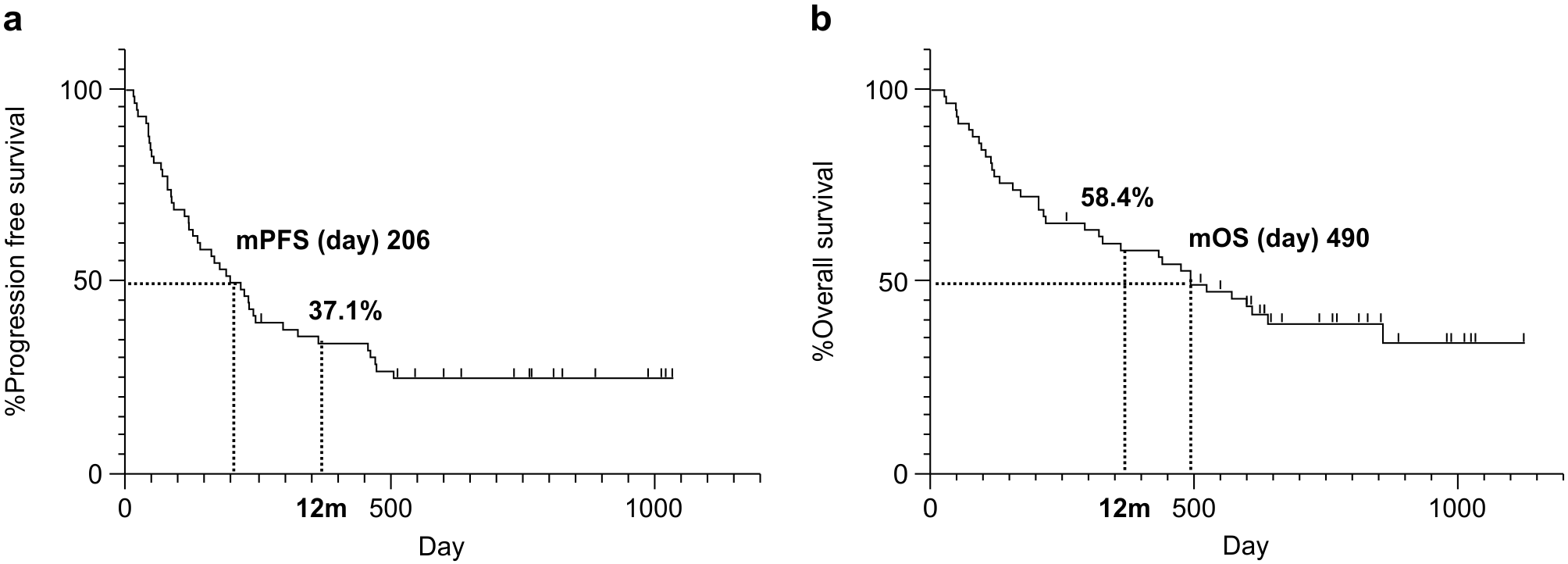

Supplement: Supplementary file 2 — Additional file 2: Supplementary Fig. 2. Overall analysis data of response to initial pembrolizumab therapy in patients with NSCLC. Analysis of mPFS (a) and mOS (b). mOS, median overall survival; mPFS, median progression-free survival; NSCLC, non-small cell lung cancer; PD, progressive disease; PR, partial response; SD, stable disease. [file 12885_2022_10445_MOESM2_ESM.tif]

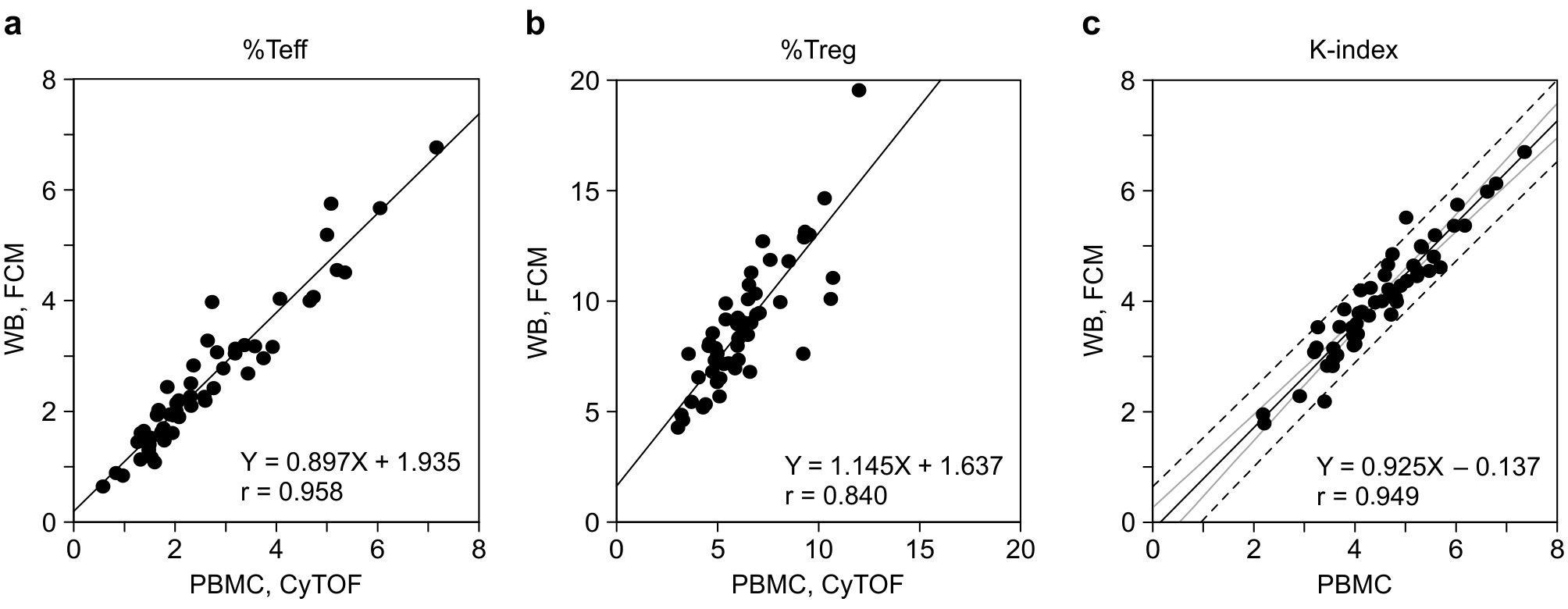

Supplement: Supplementary file 3 — Additional file 3: Supplementary Fig. 3. Correlation between measurements obtained using the PBMC and WB methods. The measurements were performed using CyTOF in the PBMC method and FCM in the WB method. The gating strategy of CyTOF analysis was performed as described in a previous study [18]. In brief, dead cells were removed using 198Pt staining. The analysis of the lower layer of CD4+ cells was the same as the FCM analysis. Blood samples were collected from 53 patients with lung cancer. (a–c) Each sample was divided into two and measured using PBMC and WB methods. Regression lines and correlation coefficients were calculated for %Teff (a), %Treg (b), and the K-index (c). The gray line represents the regression line, the light gray line represents the 95% confidence interval, and the dotted line represents the 95% prediction interval. PBMC, peripheral blood mononuclear cells; Teff, effector T cells; Tregs, regulatory T cells. [file 12885_2022_10445_MOESM3_ESM.tif]
